# The LifeStories project: Empowering voices and avoiding harm—Ethics protocol of a long-term follow-up study of individuals placed in infant care institutions in Switzerland

**DOI:** 10.3389/fpsyg.2022.1032388

**Published:** 2022-11-18

**Authors:** Patricia Lannen, Clara Bombach, Fabio Sticca, Heidi Simoni, Oskar G. Jenni

**Affiliations:** ^1^Marie Meierhofer Children’s Institute, Associated Institute of the University of Zurich, Zurich, Switzerland; ^2^Child Development Center, University Children's Hospital Zurich, Zurich, Switzerland

**Keywords:** ethics, vulnerable cohort, long-term follow-up, institutionalization, deprivation

## Abstract

Little empirical data exist to guide ethical decisions when conducting research with vulnerable populations. The current study assesses a protocol designed to mitigate risks in a population-based cohort of 246 individuals placed in care institutions as infants in a non-selective 60-year follow-up. In total, 116 (47%) individuals chose to participate, of whom 53 (55%) reported positive effects of participation such as the opportunity to fill some gaps in their life stories, to better deal with their past, and to understand previous family dynamics. Only three individuals (2.5%) explicitly reported negative short-term consequences such as feeling upset as a result of thinking about stressful times, but they nonetheless rated the usefulness of the study as high. For six participants (5%), psychological counseling sessions were initiated as a support measure. Our findings suggest that risk of harm can be managed with a rigorous ethics protocol when conducting research with a vulnerable cohort and therefore enable the voices of survivors to be heard. A step wise approach in which increasing amounts of information were presented at each step, clearly operationalized passive decline, and direct and consistent contact with highly trained staff were considered key to mitigating distress.

## Introduction

Ethics committees and funding agencies sometimes hesitate to approve research with individuals who have faced adversity, arguing convincingly that the contact might be hurtful for some individuals or even retraumatize them (Kreicbergs et al., 2004; Omerov et al., 2014; Mathews et al., 2022). Indeed, researchers must always carefully consider whether the potential benefits of conducting research with individuals that have faced adversity outweigh the costs of participation (Newman et al., 2001).

However, some have argued that the risks for individuals when sharing distressing information about their lives during data collection may be overestimated, and too little consideration is given to the benefits: talking about stigmatized and hidden experiences, contributing to society’s recognition of experiences, learning more about oneself by talking about the experience, and recognizing the need to seek help (Kreicbergs et al., 2004; Becker-Blease and Freyd, 2006; Jorm et al., 2007; Legerski and Bunnell, 2010; Sack and Ebbinghaus, 2012; Omerov et al., 2014).

Empirical evidence is paramount for developing evidence-informed ethical guidance for research with vulnerable populations. However empirical data remain scarce on how individuals who have faced adversity experience research and how well specific measures work to mitigate risks.

In this study, we present such data from a 60-year follow-up study with a cohort of individuals placed in care institutions as infants in Switzerland in the 1950s. The study was conducted as part of the Swiss National Research Program (NRP76), which analyses “the characteristics, mechanisms and effects of Swiss welfare policy and practice in their various contexts. Possible causes for welfare practices that violate and promote integrity are to be identified and the effects on those affected investigated.” (Schweizerischer Nationalfonds zur Förderung der wissenschaftlichen Forschung [SNF], 2021). This effort is part of a reconciliation process and represents a societal-level recognition of the problematic history of coerced welfare practices in Switzerland before the law reform in 1981: Many of the child welfare policies and practices in Switzerland before the law reform in 1981^1^ were rather invasive and were exercised under a legal context that sometimes threatened the integrity and basic human rights (see Hauss et al., 2018). Formally, these practices were referred to as “Compulsory Social Measures and Placements.” They included a host of measures such as placements in care institutions and foster families on farms (“contract children”; German: “Verdingkinder”) or remanding individuals in psychiatric hospitals, medication trials, forced castrations or forced adoptions. These measures served to intervene in family and personal circumstances, which, from the point of view of the authorities were slovenly (“liederlich”) or bedraggelt (“verwahrlost”) and should be “disciplined” were meant to into family and personal circumstances (see Ramsauer, 2000; Lengwiler and Praz, 2018; Unabhängige Expertenkommission, 2019). This reconciliation process was initiated after many years of survivors calling for attention and demanding that their voices be heard (Unabhängige Expertenkommission Administrative Versorgungen, 2019; Lannen et al., 2020). The authors have published an overview of the reconciliation process and how survivors have called for attention elsewhere (Lannen et al., 2020). It is one of many international reconciliation efforts related to care practices (Wright et al., 2017).

For this particular study, the NRP76 steering committee originally expressed concerns related to ethical risks and initially only approved a one-year feasibility phase pending satisfactory resolution of ethical concerns. During the feasibility phase, we developed a detailed ethics protocol to mitigate specific ethical risks for the participants. Four survivors of institutional placement, one woman and three men, aged between 54 and 60, were involved at the beginning of the research activities. Three of them had been placed in care institutions as infants between 1959 and 1965, one of them both as a toddler and an adolescent. Details of this process and its outcomes have been published elsewhere (Lannen et al., 2020). In addition, an in-depth, independent ethics review and consultation process was conducted for both the historical study of Waves 1 and 2 (Brauer, 2019a) and the current study of Wave 3 (Brauer, 2019b). Furthermore, the study was reviewed and approved by the Ethics Committee of the Faculty of Philosophy at the University of Zurich (Ref. 19.4.7). Full funding by the Swiss National Science Foundation for the study was subsequently granted.

We have opted to carefully document and publish our approach and lessons learned.

Accordingly, this paper explores the following research questions:

- How do individuals experience study participation?
- What measures are necessary, feasible, and effective to mitigate potential distress arising from research with individuals formerly placed in infant care institutions in a long-term follow-up?

We hypothesize that this 60-year follow-up with a highly vulnerable population can be implemented without causing harm using a rigorous ethics protocol.

The purpose of this paper is to strengthen the evidence-base around how vulnerable individuals experience research and to outline and evaluate a rigorous ethics protocol that has guided us through this 60-year follow-up study with formerly institutionalized individuals.

## Materials and methods

### Data base of the LifeStories project

This study uses a population-based cohort of 432 individuals born between 1952 and 1959 who have been placed in an infant care institution as a young child and have been involved at three data collection time points over a period of 60 years.

Between 1958 and 1961 (Wave 1), these individuals, then mostly under the age of three years, took part in a research study that systematically recorded their development. The study found that their physical, cognitive, social, and motor development was delayed as a result of deprivation experienced in the care institutions (Meierhofer and Keller, 1974) compared to a cohort of children from the same time and geographic location who grew up in families and were studied as part of the Zurich Longitudinal Studies (Wehrle et al., 2021).

A subsample of 143 of these institutionalized children were assessed again between 1971 and 1973 (Wave 2). The children showed increased depression, school related-problems, and stereotypies (Meierhofer and Hüttenmoser, 1975).

Between 2019 and 2021 (Wave 3), all 432 individuals that participated in the original study as infants were located through the population registry. They were invited to participate in a 60-year follow-up assessment to determine their health and well-being. The assessment used a multi-method approach involving questionnaires, neuropsychological assessments, and biographical narrative interviews (Rosenthal, 2014) to capture life trajectory.

The study is expected to improve our understanding of the long-term consequences of deprivation caused by institutionalization of small children. There are a handful of studies that look at long-term consequences of institutional placement. However, they have so far been able to follow the individuals into their 20s (Skeels, 1966; Storsbergen et al., 2010; Bick et al., 2015; Sonuga-Barke et al., 2017) or do not have early development data available for longitudinal analyses (Rushton et al., 2013).

To the best of our best knowledge, this study is therefore the longest, population-based follow-up of formerly institutionalized infants worldwide. Furthermore, since these infants were well cared for in terms of nutrition, hygiene and medical care, it provides a unique opportunity to study the long-term effects of psychosocial deprivation and disentangle from consequences stemming from physical neglect and malnutrition.

Details of the historical context of the institutionalization, the research process and study design can be found in the study protocol (Lannen et al., 2021).

### Cohort description

Of the 432 original study participants, so far, we have been able to find over 95% registered in Switzerland (*N* = 306) and *N* = 39 residing abroad through population registries. Of those identified so far, 246 were eligible and contacted for this study (as of Dec 2, 2021; the study is ongoing). Individuals were rendered ineligible for the study by these criteria:

- We were not able to find them through population registry search.
- They had a contact ban (confidentiality requirement) instated with the municipalities responsible for providing population registry information, or they were deceased.
- We found indications in the archived study materials that individuals had been adopted at an early age and contacting them would possibly disclose a previously unknown adoption.

Details of the search and eligibility have been published in the study protocol by Lannen et al. (2021). Reasons for early institutional placements included extramarital birth or single motherhood (*N* = 232, 54%), migrant status (*N* = 116, 27%), precarious living situation (*N* = 34, 8%), and other reasons, such as full-time employment of mother (*N* = 13, 3%). This information is missing for *N* = 37 (8%). The subsequent care trajectory of the children was quite heterogeneous: some stayed at the institutions for just a few months; others remained under institutional care for years. Some returned to their biological families, some were placed in foster families, and others were adopted. Many experienced multiple transitions in and out of various care contexts (Meierhofer and Hüttenmoser, 1975).

Of the 246 eligible individuals, 116 (47%) have so far decided to participate in the study. Some 51 % of the participants are female. Participants were born between 1952 and 1964, with a mean of 1957.39 (*SD* = 1.6) and a median and mode of 1958. Approximately 64 % were Swiss, 13% were citizens from other countries (11 Italian, 4 Austrian, 1 German), 20% were dual citizens (15 Swiss and Italian, 4 Swiss and German, 1 Swiss and French, 1 Swiss and Finnish), and 3% did not indicate their citizenship. Some 18% were never married, 47% were currently married or in a same-sex registered partnership, 4% were separated, 27% were divorced, and 4% were widowed. Five percent completed compulsory school, 64% completed vocational education, 8% obtained a middle school diploma, 7% had a vocational baccalaureate, and 17% held a university degree. Reports on the current occupational status showed that 39% were full-time employees, 26% were part-time employees, 3% were full-time housewives or househusbands, 25% were retired, 2% were unemployed, and 6% were not working due to disability. Twenty-two percent of the participants did not report their annual income, and of the remaining 78, 4% indicated an income of less than CHF 60.000, 37% between CHF 60,000 and CHF 90,000, 33% between CHF 90,000 and CHF 130,000, and 25% over CHF 130,000. Regression analysis showed that study participation was not selective in regards to the individuals’ development in early childhood (*B* = −0.01, *p* = 0.29) or gender (*B* = 0.08, *p* = 0.75).

### Ethical risks to this cohort

The following key ethical risks were identified for this cohort:

- It was conceivable that some individuals were unaware of their institutional placement as it took place so early in their lives (Robinson-Riegler and Robinson-Riegler, 2012; Ryffel and Simoni, 2016). There was a risk that the invitation to participate in the study would disclose a previously unknown institutional placement.
- Whereas their placement in care institutions might have been known to the individuals, the participation in the historical studies might not. This, again, might be related to their age, particularly Wave 1. It might also be because these children were reportedly subjected to many tests and interviews by a myriad of professionals on a regular basis, indistinguishable from the study in the perception of some individuals (Lannen et al., 2020). The invitation to participate in the study might disclose a previously unknown study participation.
- Some of the individuals may not have shared their history of institutional placement with their surroundings (Gabriel et al., 2021; Keller et al., 2021). The study might disclose this information to their next of kin.
- As part of the study, individuals might share personal information about their lives or view archived records that trigger emotional responses (Newman et al., 1997; Künzle et al., 2021).

### Measures to mitigate ethical risks

To mitigate the ethical risks for participants, we developed a comprehensive ethics protocol. Similar ethics protocols have been published and successfully used in research with trauma-affected individuals (Omerov et al., 2014). However, we have expanded and adapted existing approaches to create a protocol tailored specifically our target population and study context. Every step of the process and interaction with the informants was meticulously planned and prepared. According to Omerov et al. (2014), a study design that carefully considers every detail in written and personal contact reduces the number of distressed persons. In addition, researchers received extensive training to work within the framework.

The full ethics protocol can be found in the Supplementary Materials.

The ethics protocol possesses these key features:

#### Identification and search of individuals

The search for former study participants took place within a tight legal framework and in accordance with data protection laws. The procedure applied a search for individuals through the population registry. While very time consuming, this strategy minimized the risk of false identification. For details of the legal framework and search process, see Lannen et al. (2021).

#### Making contact with the cohort

We developed a graduated contact approach, with increasing amounts of information about the study provided with each step: letter 1, letter 2, phone call, personal contact, and consent form. Importantly, this approach allowed participants to control how much information they received about the study and to opt out immediately if they did not wish to learn about the study. Its aim was to avoid a sense of personal intrusion as much as possible. It also allowed the researchers to screen for any distress during gradually increasing involvement in the research process.

Direct contact with the target group was facilitated by a limited number of experienced and trained researchers throughout the research process. A lead contact researcher was assigned to each study participant and remained with them throughout the process. Withdraw at any time without explanation participants could withdraw at any. Recruitment was implemented in waves to ensure that ample time was available to communicate with each of the participants whenever necessary.

The process was set up as an opt-out process rather than opt-in, as applying an opt-in process has been shown to dramatically decrease participation rates (Beskow et al., 1990; Eilegård et al., 2013). Hence, individuals were contacted again for the next step unless they actively declined participation within a predetermined timeframe. Importantly, participation could be actively declined at any time and at each step of the process in several easy and straightforward ways: paper slip and pre-stamped return envelope, email or phone. Passive decline was also clearly operationalized as no response to final-call letter after three unsuccessful attempts to reach participant via phone. Participation was voluntary, and participants could withdraw at any time without explanation. The decline was always accepted without further probing.

The initial contact with the individuals was considered the most crucial for the ethical considerations concerning potential disclosure of institutional stay to either participants themselves or the people around them. The first letter disclosed only the year of birth rather than the full date of birth to allow for the assumption that the individual had been falsely identified. It also included a response option that allowed the individuals to state that they thought we were mistaken in our identification of them. Even though a mistaken identity was highly unlikely, because individuals were identified and sought through the population registry, the protocol planned that responses would not be challenged, and individuals would be marked as having actively declined to participate in such cases.

First, two letters were sent 2 weeks apart. Two weeks after the second letter, those who did not decline participation were telephoned by the researcher to discuss the study protocol and answer any questions they might have. Semistructured conversation guides were prepared to steer researchers through the conversations and included suggestions on how to respond to a variety of situations. Phone conversations were rehearsed in roleplay.

**Figure 1:**
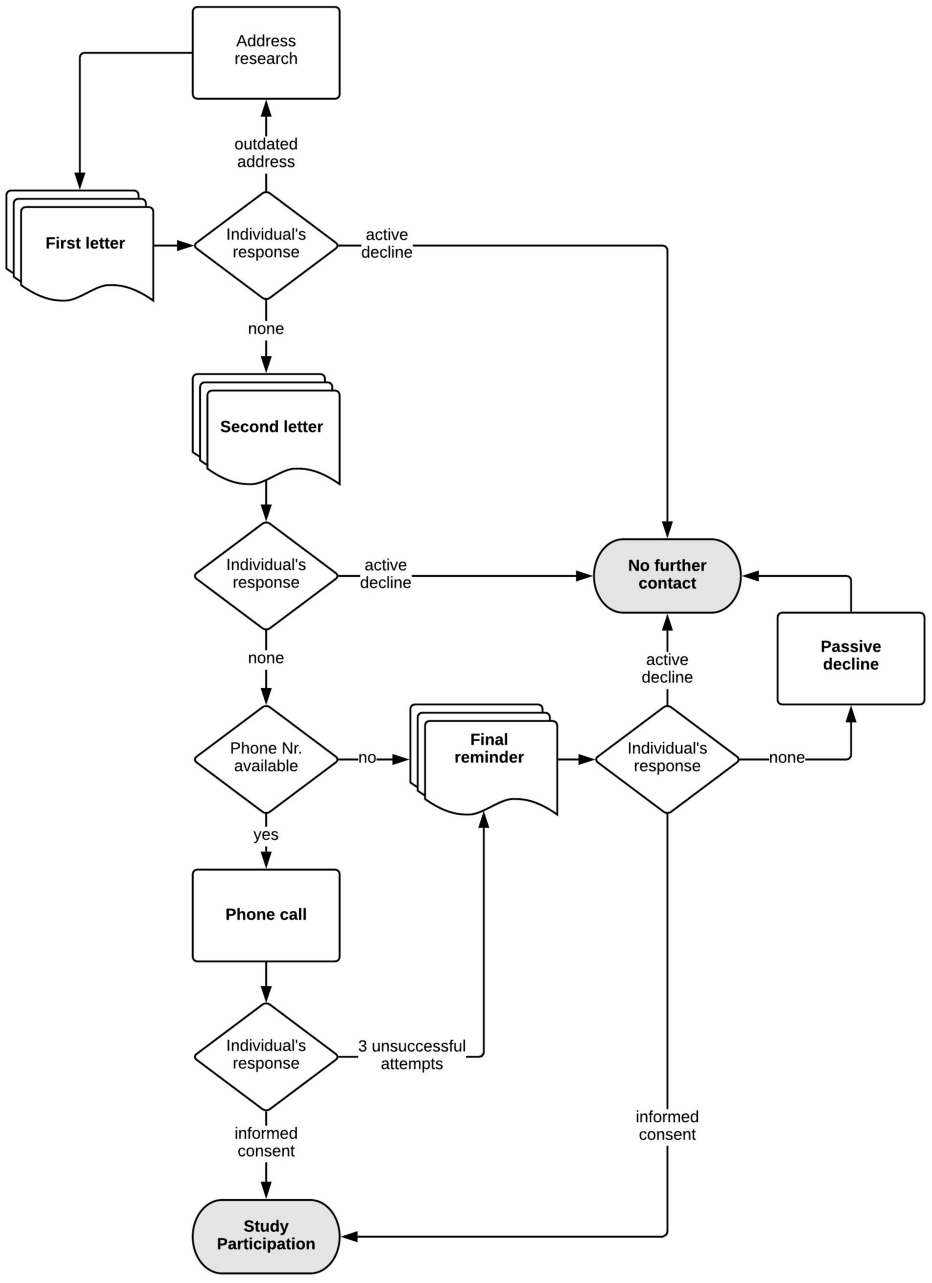
Recruitment process.

The steps in the contact process are depicted in Figure 1.

The phone conversations followed Omerov et al.’s (2013) procedure: They were structured to move from more general questions to more detailed ones to mitigate and allow assessment of potential distress and avoid a sense of personal intrusion as much as possible. When making the calls, researchers were prepared to listen at length, spoke freely and naturally without a fixed script, were emotionally available, and spoke mindfully and kindly with the individuals. The importance of this type of approach when communicating with study participants was at the core of the feedback from the survivors that were included during the preparatory phase of the study (Lannen et al., 2020). Researchers always sought verbal consent for every step of the study.

If the participant or a third party picked up the phone but told us that it was not a good time to talk, a maximum of three such calls were made. After these attempts, one last letter was sent to inform the participants that we had tried to reach them and that, if they still wished to participate in the study, they should proactively contact the study team. This final-call letter was introduced as a result of an explicit recommendation by the survivors involved during the preparatory phase of the study (Lannen et al., 2020).

After the call and with the participant’s verbal consent, a questionnaire using a battery of standardized and validated instruments related to health, well-being and the experience of study participation as well as a detailed consent form was sent to the individuals that once again explained the study purpose and what study participation would entail. It also specified the participants’ rights and the obligations of the research team, such as the fact that study participation was voluntary and consent could be withdrawn at any time without negative consequences. In addition, consent was sought from individuals to link historical and new data. Details on the questionnaire and the consent process can be found in the study protocol (Lannen et al., 2021).

If the questionnaire was not returned within 3 weeks, a reminder was sent and support with completing the questionnaire was offered. If participants still agreed to complete the questionnaire, but once again did not return it within another 3 weeks, a final reminder was sent with an abbreviated version of the questionnaire. If that questionnaire was not returned, no further attempts were made to elicit data, and these participants were coded as having passively declined in our database.

The exchanges with participants were recorded in a FileMaker database.

#### Data collection

Data collection was focused on the present health status and life trajectory during adulthood, or as far back as the participants wished to share, with no active probing of experiences related to institutionalization or the circumstances that might have led to it. The participants decided if and what they disclosed about their past experiences.

#### Remuneration

Participants were reimbursed for their efforts and received up to CHF 200 for completing the full study protocol: CHF 80 for the full questionnaire, CHF 30 if they completed the abbreviated questionnaire, CHF 80 for the neuropsychological testing, and CHF 40 for the biographical narrative interview. In addition, travel costs to the study site for neuropsychological testing and interviews were reimbursed.

#### Monitoring of distress

At the core of the ethics protocol was a tight screening framework for participants’ distress. This framework included a standardized visual-analog-scale (distress thermometer) used by the researcher and an algorithm specifying how to respond to different scenarios, including when and how to refer participants to support services (for details see data collection and instruments section).

In addition to the monitoring of distress as part of direct researcher–participant interaction, a set of items in the questionnaire asked how informants were affected by research participation (for details see data collection and instruments section). This assessment was not used to identify individuals with elevated scores. Doing so would have violated the promise to handle data anonymously. Instead, the data allowed us to monitor how the study affected the participants overall (for details see data collection and instruments section).

#### Comprehensive support system

A comprehensive set of support measures was implemented in case a participant expressed distress or the wish to talk further about past experiences as part of the exchange with the researcher.

Firstly, we had licensed psychotherapists on staff, located immediately adjacent to the study office, who could be called on for support in the event of acute distress. Furthermore, short-notice counseling and study debriefing with the psychologists on staff was available when necessary. In addition, we had access to a network of support services to which individuals could be referred. A leaflet was provided about support services, including crisis intervention and licensed external psychotherapists, self-help groups, and victim support centers. The researcher assigned to a given participant remained available for questions, concerns, and feedback throughout the study process.

In addition, study staff offered information on how to access information in the archives. During the preparatory phase of the study, we communicated with the archives and the former infant institutions to identify contact persons and inform them about the impending study. It was also possible to arrange accompaniment or debriefing after visits to the archive by a psychologist on staff if desired. Interestingly, some key feedback from survivors during the preparatory phase of the study was that while the support network was deemed crucial and much appreciated, they strongly suggested not overemphasizing its availability. They were concerned that this could be disconcerting or even trigger an exaggerated sense of impending harm as a result of study participation (Lannen et al., 2020). This mirrors findings that suggest that overly alarming language during research practice may create anxiety for participants and/or establish conditions for a self-fulfilling prophecy (Becker-Blease and Freyd, 2006).

### Data collection and instruments

The following instruments were used to collect data:

#### Participation rate

We tracked whether each decline was active or passive and its time point in a specially designed FileMaker database. This allowed us to assess whether and when each individual used the opportunity to opt-out of study participation.

#### Effect of study participation

The effect of the study on the participants was assessed with both quantitative and qualitative measures:

Standardized items to assess the effect of study participation on formerly institutionalized individuals were included at two time points: the items were included in the questionnaire and were also given to the participant to complete after the neuropsychological assessment on site.

The items were based on and adapted from a population-based study that examined the effect of study participation on bereaved parents (Dyregrov, 2004; Kreicbergs et al., 2004; Omerov et al., 2014). Items included whether study participation was perceived as useful (on a 4-point Likert scale, 0–3), whether participation had any negative effects (yes or no) or any positive effects (yes or no) on the individuals, and whether they expected effects to be long lasting (yes or no).

Many participants volunteered qualitative information on the effects of study participation during our semistructured phone conversations, when meeting with the researchers before and after the in-person data collection on health and well-being, or as part of the open-ended written comments in the questionnaire. This information was recorded systematically. Verbal consent to take notes on information related to distress and experience to participate in the study was sought during conversations that took place before formal written consent was signed, for instance, during initial phone conversations.

Furthermore, and as a standardized assessment, we used a 10-point visual-analog-scale distress thermometer to assess distress when interacting directly with the participants. This tool has been shown to be a suitable way of entering conversations with participants about distress in clinical settings and was validated with established distress measures (Mehnert et al., 2006).

**Figure 2:**
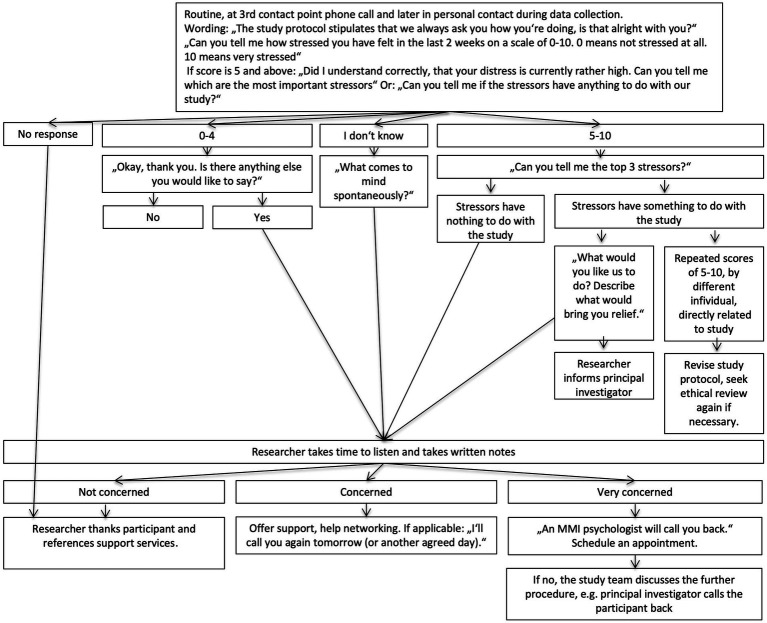
Algorithm to screen for distress.

In addition, an algorithm was created that guided researchers’ responses to the screening and semistructured responses were defined for different scores and signs of distress. The algorithm allowed standardized assessment of whether any of the elevated distress was related to the study or of other origin. The algorithm is outlined in Figure 2.

However, over the course of data collection, it became clear that the use of the distress thermometer was rather disruptive to relationship building and the rapport between researchers and participants. After initial consistent use of the distress thermometer, it was abandoned in situations when the narrative, qualitative information from the interaction was sufficient to assess distress.

We also recorded whether any steps were initiated to provide additional support.

Finally, we recorded when a study participant expressed interest in accessing the archives, what individuals told us about visiting the archives, and whether a debrief conversation about their experience at the archives took place with one of the psychologists on staff.

### Analysis

#### Participation rates

To obtain participation rates, descriptive statistics were generated using the software R software version 3.6.0 (R Core Team, 2020).

#### Effect of study participation

Descriptive analyses of the responses from the questionnaire items on the experience of study participation were run with R software version 3.6.0 (R Core Team, 2020). Specifically, we examined participants’ responses to questions about

- the usefulness of the study
- the expectation of negative and positive consequences of participating in the study in (both closed and open-end format, and
- the expectation of these negative and/or positive consequences as having long-lasting effects on their lives.

Moreover, we used *t* tests to explore whether there was an association between the usefulness of the study and the expectation of positive and/or negative long-lasting effects. Given that these data were collected both with the questionnaire and after the neuropsychological assessment with exactly the same items, the same analyses were run for both sets of data. In addition, we examined whether the mean score of the usefulness of the study changed between after completing the questionnaire and after completing the neuropsychological assessment using a *t* test for paired samples.

In addition, descriptive statistics were run for a number of individuals indicating distress as part of the interaction with researchers and for subsequent measures that were initiated.

Content analysis (Mayring, 2000) was applied to the qualitative data. Exemplary statements were extracted to illustrate and/or complement results from the quantitative assessments.

## Results

### Participation rate

Of the 246 eligible individuals, we communicated directly in one way or another with all but 31 individuals. 116 (47%) decided to participate in at least one of the study elements. Some 58 of them had participated in both Wave 1 and Wave 2 of the historic studies.

Table 1 shows the participation rate and type of decline. Some individuals chose to add written and oral comments on why they decided to decline participation. These included that they did not want to participate due to a lack of time or interest or due to health issues. Other comments stated that they did not want to go back and open up the past, but instead wanted to only move forward after difficult times. Some stated that they thought their participation would not be of use or that they did not believe in research.

**Table 1:** Participation rate and type of decline.

participated	*n* (%)	Declined	*n* (%)
Full	58 (23.6%)	Active^b^	71 (28.9%)
Partial^a^	58 (23.6%)	Passive	59 (23.9%)
Total	116 (47.2%)		130 (52.8%)

*N* = 246 eligible individuals.

a *n* = 17 declined to participate in the neuropsychological assessment, *n* = 20 declined to participate in the interviews.

b *n* = 33 declined on initial contact (letter 1), *n* = 14 after letter 2, and *n* = 24 during direct interaction with a researcher.

Three individuals (2.5%) explicitly told us that they thought we had mistaken their identity and that they were not the person we sought.

In addition, we recorded 37 partial declines. These individuals participated in at least one of the three data collection methods and hence are coded above as “decided to participate,” but declined to participate in all three study parts. Specifically, 17 individuals opted not to participate in the neuropsychological assessment, and 20 decided not to participate in the interviews.

Three individuals contacted us and requested information about accessing archives but chose not to participate in the study itself.

### Effect of study participation

#### Usefulness of the study

The usefulness of the study was measured on a scale from 0, not useful at all, to 3, very useful. The mean score was 2.27 (*SD* = 0.75) for responses to the questionnaire, and it was 2.45 (*SD* = 0.57) for responses after completing the neuropsychological assessment. This difference is statistically significant [*t*(54) = 2.11, *p* = 0.039, *d* = 0.25].

#### Positive effects

Table 2 outlines rates and types of positive effects reported by participants. Participants who reported positive effects of participation also indicated significantly higher usefulness of the study (*M* = 2.50) than did participants who did not anticipate positive effects [*M* = 2.02, *t*(79.80) = 3.14, *p* = 0.002].

**Table 2:** Reported positive effects of participation.

	After questionnaire *n* (%)	After neuropsych. Assessment *n* (%)
Long-lasting positive effect	35 (30%)	34 (34%)
Short-lasing positive effect	18 (15%)	9 (9%)
	**After questionnaire *n* (%)**	**After neurol. Assessment *n* (%)**
Long lasting positive effect	35 (30%)	34 (34%)
Short-lasting positive effect	18 (15%)	9 (9%)

*N_questionnaire_ = 116, N_neuropsych. assessment_ = 99.*

Some of the positive effects individuals reported included the following:

They thought that the study provided an opportunity to fill some gaps in their life stories, which they did not remember due to their age at the time they were in an institution. One participant, for example, stated: “Finally, the first years of my life are documented. It feels like my life story is being completed, who I am.”

Some also reported that participating in the study helped them to better understand what happened at the time, their family dynamics, and how their life had developed. Individuals reported that participating in the study provided an opportunity to deal with their past. It allowed them to reflect on their biography, draw conclusions, and think about how events were linked. Some reported that it even helped them deal with the past and heal some of their wounds. They reported that they were taking stock of their lives and thought about their future as well. Several individuals felt that it made them think about everything they had achieved in their lives and made statements such as: “The more I process and let go, the freer I am today as a grown woman.”

Several participants reported that they had never or rarely talked about their past and that they were grateful for the opportunity to finally do so. This was expressed, for example, in the following statement: “For a long time, it was not possible to talk about my childhood. In small steps, I have tried over the last 7 years, and tried again, and finally, as part of the study, I was successful.”

Others reported that participating in the study created an opportunity to talk about the past within the family, with their siblings, and in some cases also with their parents. Individuals also indicated that they felt gratified to contribute to the historical reappraisal and reconciliation process and enable a better understanding of what happened in relation to historical institutional placements in statements such as: “I’m sure that my story contributed a tiny bit to mending past mistakes. If that’s the case, that makes me so happy!”

Quotations are open-ended comments collected as part of the questionnaire survey from two study participants.

#### Negative effects and distress

Only three individuals reported negative consequences on the questionnaire. However, none of the three expected the negative consequences to be long lasting. All three also reported positive consequences, and one expected these consequences to be long lasting. Two of these individuals who had reported negative consequences also provided a rating on the usefulness of the study, and both gave the highest score. In the open-ended question about the negative effects of study participation, the participants noted that describing the difficult years was upsetting and distressing but also emphasized that it felt good to make a personal contribution to the study.

For six individuals (5%), the researchers decided to initiate a counseling session with the in-house psychology team. All except one of these individuals were referred after viewing the archived information.

As mentioned, the distress thermometer was used in the first phase of the study. Data are available for 80 individuals in total. As the distress thermometer was used in multiple interactions, 145 data points are available for these 80 individuals. Seven of the 80 participants for whom data is available indicated elevated distress, represented by a score between 4 and 8 out of 10, that was directly related to the study: three individuals were upset after thinking about the past; one individual was upset because she felt that while the study in the 1950s might have benefited later children, she herself did not receive any support at that time; one person indicated that conversation in their family was triggered and they felt that, other than their spouse, nobody seemed to be interested in their past; one person reported being upset after viewing the archive, and one person was distressed by the study uncovering their institutional placement. Other individuals indicated distress that was unrelated to the study.

### Disclosure of institutional stay

Particularly noteworthy were those 11 who reported that they did not know about their institutional placement prior to the study. Eight of these chose to participate. Generally, individuals reported a combination of distress about their changed life stories, but at the same time relief at knowing.

### Distressed nonparticipants

During the course of the study, three participants that we coded as distressed nonparticipants reached out to us. One participant contacted the study team by phone and initially was quite upset. Their researcher was able to respond with both empathy and information related to specific concerns about data protection and privacy and therefore calm the situation. Although the individual declined participation, they were interested in information concerning access to the archives. Two additional participants relayed one voice message and one written message from which we concluded that the participants were likely distressed as a result of being contacted. Although both participants thanked us for conducting the study, they vigorously requested that they not be contacted again, upon which we did not follow up to discuss specific concerns or reasons for distress. Both indicated in their messages that being contacted brought back distressing memories.

## Discussion

This study describes and assesses a comprehensive ethics protocol for conducting research with a vulnerable cohort, namely a 60-year follow-up with individuals that were placed in infant care institutions under conditions of socio-emotional deprivation during early childhood in Switzerland in the 1950s. The study also presents empirical data on the effects of study participation on the individuals.

In this study, almost half of eligible individuals agreed to participate. This lies somewhat below the response rate of the Zurich Longitudinal Studies, where it was some 60% (Wehrle et al., 2021). Unlike the institutionalized individuals, participants of the Zurich Longitudinal Studies were involved continuously in data collection until age 18, and their children have since also been involved in another cohort study (personal communication with the authors). However, the recruitment success in our study exceeded our expectations, given the 50 to 60-year period between the first assessment time point and this most recent one.

The stepwise approach that we developed to contacting individuals relies on the fact that individuals are able to determine when something is too much and will overwhelm them (Black and Black, 2007). However, individuals may differ in the threshold of distress and the amount of information they need to reach an informed decision on whether to participate. If the information is introduced slowly, those approached have an opportunity to be mindful of when information starts to feel overwhelming and decide to withdraw. It allows them to make a careful assessment of whether the study is something they want or could engage in. There are often implicit assumptions that survivors are not emotionally stable enough to assess risk or seek help (Black and Black, 2007). However, our findings are consistent with evidence that suggests that participants can and do decline to participate when they are concerned about becoming upset (Brabin and Berah, 1995; Jorm et al., 2007; Dyregrov et al., 2011b; Omerov et al., 2014). It is therefore important to consider that participation might have caused distress for those who have declined and highlights the importance of Omerov et al.’s (2014) recommendation to accept decline immediately without further probing.

Providing detailed information on what will take place as part of study is consistent with the American Psychological Association (APA), 2017 Ethics Code (Standard 8.02) that concerns about upsetting participants can be addressed with informed consent that allows participants to refuse or to end participation in the research. Appropriate informed consent allows potential participants to decide whether participation is in their best interest and whether it involves risks they are willing and able to take (Black and Black, 2007).

Some individuals stated that they did not know that they had been placed in an institution. They did not remember the placement due to their age at the time (Robinson-Riegler and Robinson-Riegler, 2012; Ryffel and Simoni, 2016). Also, institutional placement practices at the time were often accompanied by a burden of silence and stigma (Unabhängige Expertenkommission Administrative Versorgungen, 2019), and many survivors reported that, as a result, the topic was not discussed in the family (Sack and Ebbinghaus, 2012; Ryffel and Simoni, 2016; Lengwiler, 2018). The risk of disclosing the institutional placement by the study had to be considered carefully, as it bears the risk of causing distress through the disruption of individuals’ coping strategies, sense of coherence, and biographical narrative (Antonovsky, 1987; Ryffel and Simoni, 2016). However, while having their institutional placement disclosed was stressful for some, for others, the disclosure brought a sense of relief: injustice and the taboo that surrounds it cannot be uncovered without providing a voice to those affected. Or as Becker-Blease and Freyd (2006) put it: “To the extent that silence is part of the problem—silence impedes scientific discovery, helps abusers and hurts victims—then this is no trivial matter.”

We had also expected possible distress from a disclosure of unknown participation in research. We assumed that there might be concerns related to the nature of the historical study procedures, given the many ethically challenging studies conducted in medicine and psychiatry during the same era as Wave 1 (Germann, 1997; Rietmann et al., 2018). While the majority of individuals did in fact not know that they were included in a research study at a young age, this was never discussed as a source of distress. Similarly, there were no reports of unintended disclosure of institutional stay to a next-of-kin. While the majority of individuals did not know that they were included in a research study at a young age, this was never discussed as a source of distress. It would have been conceivable that learning about being involved in a research study as children might trigger unease or even distress.

Some individuals decided to only take part in a selective type of assessment method. This result is relevant in the context of research that has shown that different assessment methods seem to cause different levels of distress to different individuals (Legerski and Bunnell, 2010). Legerski and Bunnell (2010) also concluded that participation in multiple survey methods did not increase distress but raised greater perceived personal benefits and led participants to rate the overall experience of participating in research more positively. They concluded that methods of assessment may influence how positively or negatively individuals experience the research process. Hence, using multiple data collection methods not only enriches the data set with a broad data source but also allows individuals the choice of assessing the potential limits of their distress tolerance while still contributing to the study.

A little over half of the participants reported positive effects of participating in the study. The positive effects that were reported are in line with effects reported in other studies such as a sense of helping others, learning more about oneself by talking about the experience, providing a helpful review of life events, increasing self-awareness, reducing self-perceptions of blame, clarifying memories, generating feelings of relief, and facilitating recall of positive aspects of life, talking about stigmatized and hidden experiences, contributing to society’s recognition of experiences, and recognizing the need to seek help (Disch, 2001; Carlson et al., 2003; Kreicbergs et al., 2004; Becker-Blease and Freyd, 2006; Jorm et al., 2007; Legerski and Bunnell, 2010; Sack and Ebbinghaus, 2012; Omerov et al., 2014). It is noteworthy that several of the comments on positive effects were related not only to study participation *per se* but also to the opportunity to view archived materials. Many individuals that were placed in institutions do not have access to any documentation or memory of these early years. For many, accessing photographs, descriptions of their early environment, and daily routines was possible now thanks to being invited to participate in the study. It allowed them to add an important piece to the stories of their lives, for which many expressed gratitude. Many cases are documented in other studies where individuals report that only reviewing the files provides a sense that memories have found their place in their stories (Furrer, 2018). The seminal work of Aron Antonovsky on salutogenesis describes this very “sense of coherence,” the degree to which one perceives life’s events as comprehensible, manageable, and meaningful, to be crucial to successfully coping with them and living a healthy life despite adversity (Antonovsky, 1987).

In addition, three individuals who participated in the study reported negative effects as part of our the standardized assessment on how individuals experienced study participation. They thought that completing the questionnaire brought back memories of difficult times. All three individuals who reported negative effects of the study thought that the effects were short lived. Becker-Blease and Freyd (2006) stated that even when negative feelings are evoked by participating in a study, these are not necessarily an indication of psychological harm. Feelings such as grief, anger, and fear in response to remembering past events may be a transitory negative state that is understandable but not necessarily harmful. Newman and Kaloupek (2004) reported that if the level of emotional distress during participation is manageable for the individual, it may reflect emotional engagement in the research project rather than indicating harm. Furthermore, Edwards et al. (2009) found that negative emotional responses experienced during their study were associated with perceived personal benefits from participation. This is consistent with a number of trauma-related studies that suggest that a minority of participants becomes distressed as a result of data collection and that the distress diminishes quickly and is unlikely to cause retraumatization or long-term harm (Runeson and Beskow, 1991; Dyregrov et al., 2000, 2011b; Dyregrov, 2004; Galea et al., 2005; Becker-Blease and Freyd, 2006; Jorm et al., 2007; Legerski and Bunnell, 2010; Eilegård et al., 2013; Omerov et al., 2014). This is consistent with the formal definition of minimal risk (U.S. Department of Health and Human Services, 2018). Interestingly, the prevalence of negatively affected individuals in a study cannot be fully explained by answering questions about adverse events, as Omerov et al. (2014) showed by using a unaffected control group. He found that even in the unaffected control group, with no questions related to an adverse event, some individuals reported being negatively affected by completing the questionnaire. This finding was confirmed by other research (Jorm et al., 2007). However, other studies report that of the individuals who do report some distress, only a few experiences severe distress (Kreicbergs et al., 2004; Mathews et al., 2022). These individuals are obviously of great concern, and health care services must be readily available to provide support (Becker-Blease and Freyd, 2006).

Importantly, all three participants who reported negative effects of the study also reported positive effects. And two individuals for whom data are available still thought that study participation was useful. This is consistent with findings from other studies; for example, Omerov et al. (2014) reported that 50% of suicide-bereaved parents who were negatively affected by study participation also said that they were positively affected. Kreicbergs et al. (2004) state that 99% (*N* = 423) of parents who lost a child to cancer reported that that study was valuable. Of these, 68% were positively affected by their participation, and 28% were negatively affected.

It is noteworthy that perceived usefulness of the study increased from the time participants completed the questionnaire to after the on-site assessment. We conclude from statements of the participants that this was a result of additional conversations between researcher and participants. Hence, using highly trained staff that remains consistent throughout the study and that is able to identify distress and respond skillfully proved to be very important. This is consistent with Omerov et al. (2014), who stressed the importance of a single personal contact following informants throughout the study.

The importance of the quality of interaction and empathy that participants fed back to us, was also one of the key findings from the preparatory phase of the study with survivors (Lannen et al., 2020). However, Josselson (2007) describes the role of the researcher as dual, as an “intimate relationship with the participant” combined with a “professional responsible role in the scholarly community.” The responsibility researchers have to co-construct the relationship requires them to continuously reflect on their parts in the exchange. It requires regular reflection on the limits and possibilities within a study context in terms of expectations and wishes that study participants may have. Nonetheless, we infer that the relationship between researchers and participants can play an important role in mitigating distress when doing research with individuals that have faced adversity. This was tangibly reflected in a quotation by one study participant who had taken up the offer to complete the questionnaire jointly with the researcher: “I was by myself, at home, trying to fill in the questionnaire. And I could not do it. It felt overwhelming. It was too painful. But here, with you, with someone who cares and listens, it still hurts. But it’s ok. The study is important, and with you, here, I can do it and make a contribution.”

The study has several strengths and limitations: Strengths include the population-based nature of the study. It also includes the search strategy through population registry to avoid false identification as part of this follow-up and related success in finding many of the former study participants 60 years later. In addition, over 50% of individuals participated in the study 60 years later and participation was found to be non-selective in terms of key criteria. Limitations include the fact that we do not have information on distress from all of the individual we contacted. However, there were only few individuals with whom we were not in touch at all. A second limitation is that we discontinued our standardized distress screening (distress thermometer) midway through the study. This decision was made because preliminary analysis of qualitative data had shown the important mitigating effect of relationship building on distress. Researchers had felt that the standardized screening was disruptive to relationship-building. As a result, we abandoned the standardized distress screening as part of the interaction with the participants, but continued to screen participants for distress qualitatively as part of each conversation.

In conclusion, the ethics protocol presented here provided an opportunity for those who wished to have their voices heard while carefully screening for and supporting those who experienced distress as a result of participating in the study.

Sack and Ebbinghaus (2012, 350) show that the primary wish of formerly institutionalized individuals is that “negative experiences of institutionalization are recognized by today’s society as an injustice. This is the only way to overcome the sense of stigmatization of ‘having been an institutionalized child and neglected by society’ that still lingers.” The direct voices of those that participated in the historical studies could not be captured at that time due to their age. However, study participation has given these individuals another opportunity to speak out and share their stories. In fact, “it is by rehabilitation and recognition of past injustice through society that victims receive a voice” (Matter, 2018: 332 f.; Ziegler et al., 2018: 12).

## Data availability statement

The raw data supporting the conclusions of this article will be made available by the authors upon request, without undue reservation.

## Ethics statement

This study involving human participants was reviewed and approved by the Ethics Committee of the Faculty of Philosophy of the University of Zurich. The participants provided their written informed consent to participate in this study.

## Author contributions

PL is the principal investigator of the study, conceptualized and wrote the original draft of the paper, and incorporated co-author and reviewer feedback. CB is a senior researcher in the project, contributed to the conceptualization of the paper, reviewed the qualitative analysis and results, and made writing contributions to all sections of the paper, particularly the introduction and discussion. FS is a senior researcher in the project, conducted the analysis of the quantitative data, and reviewed the analysis and results section related to quantitative data analysis. HS and OJ are co-investigators of the study and reviewed the final version of the paper. All authors contributed to the article and approved the manuscript.

## Funding

The project was funded by the Swiss National Science Foundation, under the umbrella of the National Research Programme 76 Welfare and Coercion (SNF 407640_177394); it was co-funded by the Maiores Foundation. Additional funds to expand the interview part of the study were provided by the Lotteriefonds of Zurich and the City of Zurich. A contribution to the publication was made by the Grueniger Foundation.

## Conflict of interest

The authors declare that the research was conducted in the absence of any commercial or financial relationships that could be construed as a potential conflict of interest.

## Publisher’s note

All claims expressed in this article are solely those of the authors and do not necessarily represent those of their affiliated organizations, or those of the publisher, the editors and the reviewers. Any product that may be evaluated in this article, or claim that may be made by its manufacturer, is not guaranteed or endorsed by the publisher.

## Footnotes

^1^The Swiss Civil Code, introduced in 1912, allowed access to "conduct that is assessed as deviating from the norm" and granted authorities considerable leeway in their interpretation and justification of the removal and placement of a person (*cf.* Seglias, 2018, p. 23). In 1981, this law was amended, thus fulfilling an important requirement of the UN Convention on Human Rights (see Seglias, 2018, p. 31).
