# Supplementary material for: The LifeStories project: Empowering voices and avoiding harm—Ethics protocol of a long-term follow-up study of individuals placed in infant care institutions in Switzerland
Source: Front Psychol. 2022 Nov 18;13:1032388. doi: 10.3389/fpsyg.2022.1032388 (PMC9718425; doi:10.3389/fpsyg.2022.1032388)
Supplement: Supplementary file 1 [file Data_Sheet_1.pdf]

Supplementary Material

1 Ethics Protocol

1.1 Life stories over 60 years—Institutional placement of young children (*1953–1959)

1.1.1 Preparation

- The procedure for finding potential study participants operates within a legal framework.
- The research team is aware of the current research in the field of compulsory social measures in Switzerland, and research on potential impact of an institutional upbringing and experiencing adverse life events.
- A support system is developed that includes easy access to psychologists internally and a leaflet of counseling institutions (e.g. psychological, victim assistance, and self-help and support groups) and archives. The research team also has access to supervision and psychological support throughout the study.
- The research team signs confidentiality agreements.
- A password-protected database is created which tracks the contact points and interactions with the study participants. Access is restricted to the core research team.
- All folders used on the internal server to store confidential information about the project are password-protected and are only accessible to the core research team. Those researchers that are in direct contact with the study participants do not have access to the historical data on that participant.
- The research team is trained and prepared for interaction with the study participants as well as for data collection through questionnaires, interviews, and neuropsychological testing. The research team makes time and is prepared for possibly long conversations with study participants.
- Relevant documents, including letters, procedures, and information about the study, were developed in collaboration with people who had themselves been placed in infant care institutions.
- A step-wise approach to contacting individuals is implemented, with increasing information about the study at each step to provide the participants with an opportunity to opt out without being overwhelmed as far as possible. Enough time is left between contact steps for the individuals to respond.
- A lead contact person from the research team remains the consistent point of contact for a given study participant throughout the duration of the study.
- Multiple ways to contact researchers: phone, text, email, and mail return slip.
- Researchers are prepared to screen distress in every interaction with the participants, when possible on a standardized tool, and to use an algorithm that guides how they respond in case of elevated distress. If elevated stress values directly related to the study occur repeatedly, the study protocol is reassessed.
- Exclusion criteria are carefully defined.
- Passive decline is clearly operationalized for a number of scenarios.
- Possible scenarios, including reactions, questions, information, and emotions, are rehearsed in advance within the team and under the supervision of the senior staff member.

- Eligibility criteria for individuals to be contacted are clearly defined to avoid harm for instance for those presumed to have been adopted without their knowledge, or those who had declined participation in a preparatory study in the past.
- Initiating contact procedure is avoided during regional holidays (summer or winter holiday season).

1.1.2 Making contact by mail (letters)

- Letters are sent out in waves over several months to ensure study staff have the necessary time to communicate promptly with study participants.
- The first letter only includes minimal information about the study and minimal identifying information.
- The second letter includes detailed information about the study and announces the upcoming telephone contact. There is a possibility to specify a preferred phone number and time slot or to choose not to be contacted via phone and still participate in the study by communicating only in writing.
- Researcher phone number, email, and postal address is evident throughout in letters and on the website.
- The research team is set up in a way that provides the participants with the possibility to choose between a male and female researcher and to communicate in their mother tongue.
- Opting out of the study is possible at any time and via return mail slip, email, or phone without the need to specify a reason.
- The check boxes on the return mail slip include the option "I am not the person you are looking for."

1.1.3 Phone contact

- A telephone protocol is developed to guide the research team when making contact. The protocol starts by providing an opportunity to ask questions and then moves from general towards more specific information on the study. The researcher goes through the consent form for a first time; a detailed consent form is later sent in writing and the participant asked to sign if they choose to participate.
- If someone other than the target person answers the phone, the study is portrayed as a general study on health and well-being to avoid disclosing the target person's institutional history to someone who might not know about it. The researcher asks to be able to call back at a different time.
- Reference is always made to the fact that the research team is and remains available to assist with queries and refers to counseling.
- If the time of contact is not suitable, a new date is arranged. If this happens three times, a final call letter is sent, and nonresponse is considered to indicate that the target person has passive declined participation.
- If certain times have been scheduled with participants, calls are made at these times.
- Researchers do not further probe or inquire when someone declines participation. There is an optional field in which reason for declining can be specified.
- The option to end participation without explanation and without any consequences at any time during the study is repeated.

1.1.4 Data collection

- A detailed informed consent form is provided and signed by participants before data collection begins.
- The questionnaire battery includes questions about negative and positive effects of the study and its perceived benefits to obtain an overall sense of how study participation affects the cohort.
- Psychologists are available on site should the study participant or the researcher need support.
- During data collection, no questions refer directly to difficult or stressful experiences. If a person chooses to talk about such experiences, the researcher takes time to listen and responds with empathy

1.1.5 After data collection

- The research team remains available.
- Regular information on the status of the study is provided in a newsletter by email or mail, as previously agreed with the study participants.
- The website is regularly updated with current information on the study.
- The results of the study are made available to the study participants free of charge. They are invited to public events on the project.
